# iNOS activity is critical for the clearance of *Burkholderia mallei* from infected RAW 264.7 murine macrophages

**DOI:** 10.1111/j.1462-5822.2007.01063.x

**Published:** 2007-10-28

**Authors:** Paul J Brett, Mary N Burtnick, Hua Su, Vinod Nair, Frank C Gherardini

**Affiliations:** 1 Laboratory of Zoonotic Pathogens NIAID, NIH, Hamilton, MT 59840, USA; 2 Research Technologies Section, RTB, Rocky Mountain Laboratories NIAID, NIH, Hamilton, MT 59840, USA

## Abstract

*Burkholderia mallei* is a facultative intracellular pathogen that can cause fatal disease in animals and humans. To better understand the role of phagocytic cells in the control of infections caused by this organism, studies were initiated to examine the interactions of *B. mallei* with RAW 264.7 murine macrophages. Utilizing modified kanamycin-protection assays, *B. mallei* was shown to survive and replicate in RAW 264.7 cells infected at multiplicities of infection (moi) of ≤ 1. In contrast, the organism was efficiently cleared by the macrophages when infected at an moi of 10. Interestingly, studies demonstrated that the monolayers only produced high levels of TNF-α, IL-6, IL-10, GM-CSF, RANTES and IFN-β when infected at an moi of 10. In addition, nitric oxide assays and inducible nitric oxide synthase (iNOS) immunoblot analyses revealed a strong correlation between iNOS activity and clearance of *B. mallei* from RAW 264.7 cells. Furthermore, treatment of activated macrophages with the iNOS inhibitor, aminoguanidine, inhibited clearance of *B. mallei* from infected monolayers. Based upon these results, it appears that moi significantly influence the outcome of interactions between *B. mallei* and murine macrophages and that iNOS activity is critical for the clearance of *B. mallei* from activated RAW 264.7 cells.

## Introduction

*Burkholderia mallei* is a non-motile, facultative intracellular, Gram-negative bacillus that causes a debilitating disease known as glanders. This zoonotic pathogen is an obligate animal parasite that is primarily responsible for disease in solipeds and occasionally other mammals, including humans (Howe and Miller, 1947; Redfearn *et al*., 1966; Yabuuchi *et al*., 1992; Srinivasan *et al*., 2001). In regions where glanders remains endemic, chronically infected horses are the only known reservoir of this bacterial pathogen. Human infections, although rare, are thought to be acquired via the inoculation of mucocutaneous tissues with aerosols or secretions from diseased animals. The clinical progression of human glanders is similar to that observed in solipeds and may manifest as chronic or acute localized infections, acute pulmonary infections or fulminating septicemias. In the absence of chemotherapeutic intervention, glanders is invariably fatal (Wilkinson, 1981; Dance, 1996; Bartlett, 2005). Because of this, the Centers for Disease Control and Prevention currently list *B. mallei* as a category B biothreat agent (Rotz *et al*., 2002).

Studies have demonstrated that *B. mallei* expresses a number of important virulence determinants that are required for survival in various animal models of infection. Included among these are a capsular polysaccharide, the Bsa type III secretion system (T3SS), a type VI secretion system and the VirAG two-component gene regulatory system (DeShazer *et al*., 2001; Nierman *et al*., 2004; Ulrich and DeShazer, 2004; Schell *et al*., 2007). Recently, studies have demonstrated that the T3SS expressed by this organism is important for survival within J774.2 murine macrophages (Ribot and Ulrich, 2006). Consistent with these findings, it has been shown that *B. mallei* utilizes this T3SS in order to facilitate early vacuolar escape following internalization by the macrophages (Ribot and Ulrich, 2006). Interestingly, it also appears that the T3SS is necessary for intra- and intercellular actin-based motility, presumably by facilitating the ability of *B. mallei* to access intracellular pools of actin (Stevens *et al*., 2005; Ribot and Ulrich, 2006; Schell *et al*., 2007). At present, however, little else is known about the molecular mechanisms utilized by this organism to persist within phagocytic cells or to evade specific host defence responses.

Macrophages play an integral role in the development of innate and adaptive immune responses against bacterial pathogens. Following stimulation of macrophages by pathogen-associated molecular patterns (PAMPs), pattern-recognition receptor (PRR) signalling culminates in the induction of a variety of bactericidal responses (Barton and Medzhitov, 2003; Miggin and O'Neill, 2006). Two of the most potent mechanisms utilized by activated macrophages to kill bacteria involve the production of reactive oxygen species (ROS) and reactive nitrogen oxide species (RNOS) (Coleman, 2001; Vazquez-Torres and Fang, 2001; Fang, 2004). Inducible nitric oxide synthase (iNOS) is responsible for generating high levels of nitric oxide (NO) in activated macrophages. In biological systems, RNOS are then formed by the rapid oxidation of NO to reactive species such as nitrogen dioxide and dinitrogen trioxide (MacMicking *et al*., 1997; Coleman, 2001; Fang, 2004). Under conditions favouring the production of high concentrations of both NO and superoxide anion, the highly reactive peroxynitrite anion is also generated. In activated macrophages, RNOS play an important role in the clearance of intracellular bacteria due to their cytotoxic effects (MacMicking *et al*., 1997; Coleman, 2001). At low concentrations, RNOS are unstable and rapidly nitrosate amines and thiols, whereas at higher concentrations, they induce lipid peroxidation as well as nitrosate iron-/sulfur-containing reactive centres, nucleic acids and tyrosine residues (Coleman, 2001; Fang, 2004).

Many intracellular bacteria have developed mechanisms to avoid killing by phagocytic cells. Some of these include the ability to prevent phagosomal maturation, phagosomal lysis, phagosomal acidification, and inhibition of the respiratory burst, as well as the production of detoxifiers that can counteract the bactericidal effects of ROS and RNOS (Collins and Kaufmann, 2001; Smith *et al*., 2001; Fang, 2004). Recently, studies have demonstrated that, following uptake by a variety of murine macrophage cell lines, *Burkholderia pseudomallei* and *B. cepacia* were poor activators of iNOS expression and NO production respectively (Saini *et al*., 1999; Utaisincharoen *et al*., 2001). Based upon these observations, it has been proposed that these organisms may be capable of interfering with RNOS responses in order to promote their survival within phagocytic cells. To date, few studies have examined the survival characteristics of *B. mallei* within phagocytic cells. In the present study, we utilized a combination of cellular, immunological and biochemical approaches to investigate the interactions of this organism with RAW 264.7 murine macrophages. In addition, we assessed the importance of iNOS activity with regard to these interactions.

## Results

### Influence of multiplicity of infection (moi) on *B. mallei* interactions with RAW 264.7 cells

**Fig. 1:**
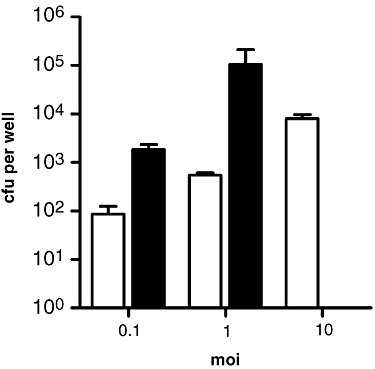
Survival characteristics of *B. mallei* in RAW 264.7 cells. Monolayers were infected with *B. mallei* at moi ranging from 0.1 to 10. Uptake (white bars) and intracellular survival (black bars) were quantified at 3 and 24 h post infection respectively. Values represent the means ± SD of three independent experiments.

Previous studies have shown that a number of pathogenic *Burkholderia* species, including *B. pseudomallei* and *B. cenocepacia*, are able to survive and replicate within RAW 264.7 murine macrophages (Jones *et al*., 1997; Miyagi *et al*., 1997; Utaisincharoen *et al*., 2001; Lamothe *et al*., 2007). To examine the ability of *B. mallei* to survive within this cell line, bacterial uptake and intracellular survival phenotypes were characterized utilizing modified kanamycin-protection assays. To optimize initial assay conditions, monolayers were infected with *B. mallei* at moi ranging from 0.1 to 10. As would be predicted, results demonstrated that uptake of *B. mallei* by the monolayers increased in a dose-dependent fashion (3 h; Fig. 1). Curiously, *B. mallei* was shown to survive and replicate in RAW 264.7 cells infected at moi of ≤ 1, but not at an moi of 10 (24 h; Fig. 1).

**Fig. 2:**
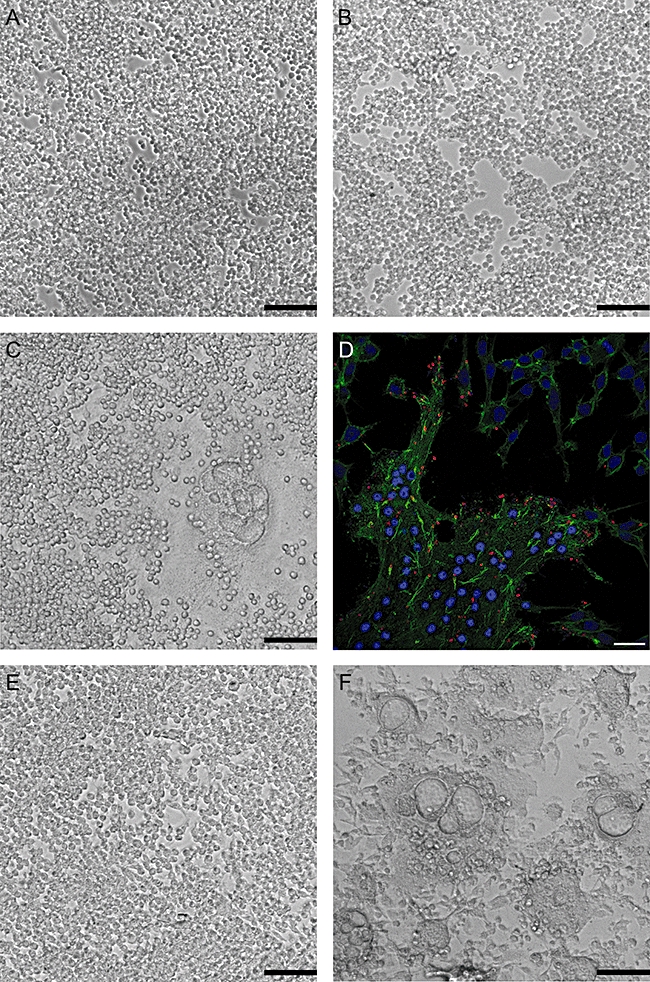
Microscopic analysis of RAW 264.7 cells infected with *B. mallei* and *B. pseudomallei*. Monolayers were fixed and examined at 24 h post infection. Light micrographs: (A) mock infected, (B) *B. mallei* moi 0.1, (C) *B. mallei* moi 1, (E) *B. mallei* moi 10, and (F) *B. pseudomallei* moi 10. Black scale bars = 1 mm. D. Confocal micrograph of RAW 264.7 cells infected with *B. mallei* at an moi of 1. Bacteria were stained red with rabbit anti-*Burkholderia thailandensis* polyclonal sera and anti-rabbit IgG Alexa Fluor® 568, actin was stained green with Alexa Fluor® 488 phalloidin, and nuclei were stained blue with DRAQ5™. White scale bar = 20 μm. Micrographs are representative of at least three independent experiments.

**Fig. 3:**
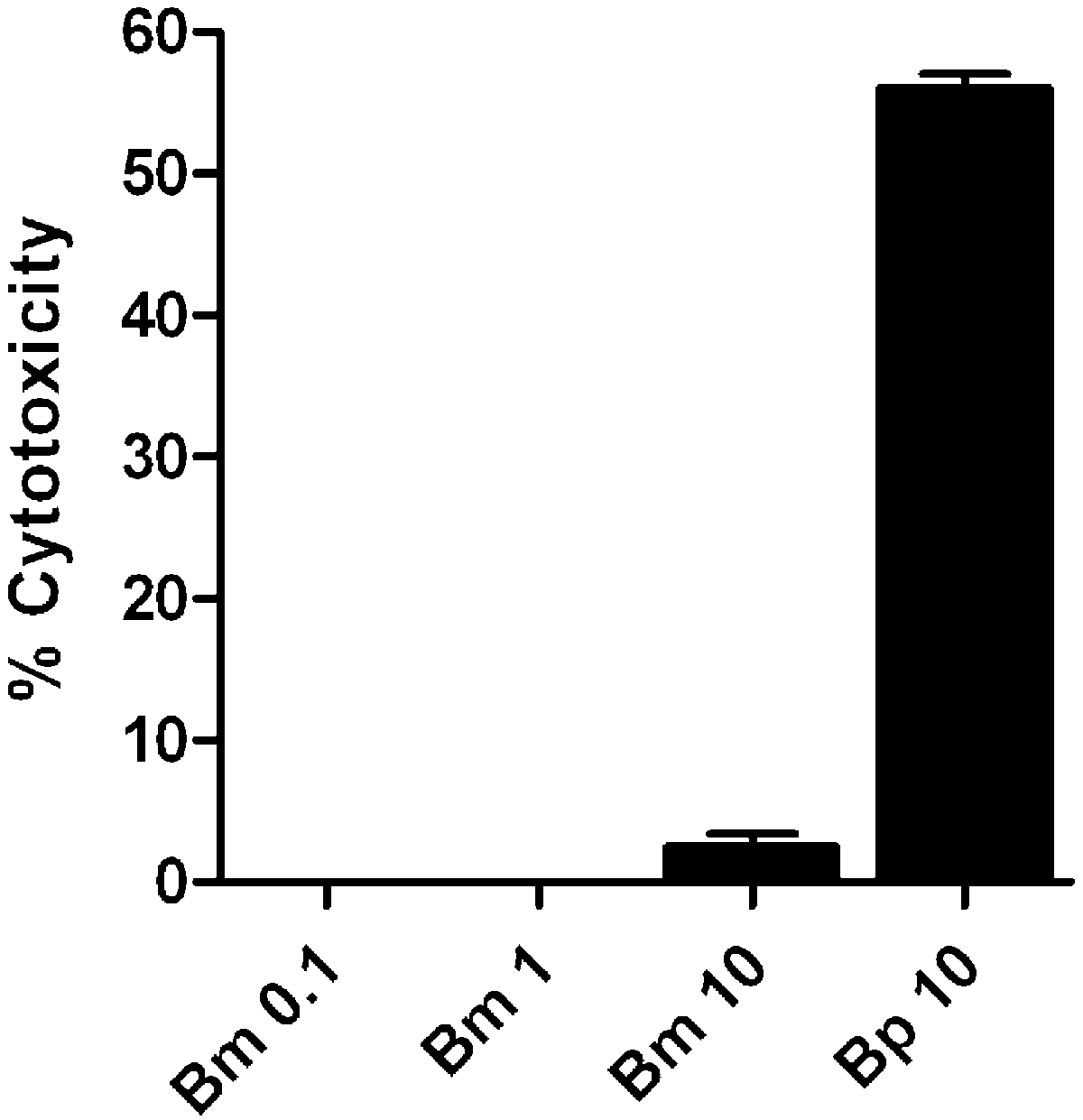
Cellular integrity of RAW 264.7 cells infected with *B. mallei* and *B. pseudomallei*. Monolayers were infected with *B. mallei* at moi 0.1 (Bm 0.1), moi 1 (Bm 1), moi 10 (Bm 10) and *B. pseudomallei* at moi 10 (Bp 10). Per cent cytotoxicity was determined by assaying for LDH release in culture supernatants at 24 h post infection. Values represent the means ± SD of three independent experiments assayed in duplicate.

Several studies have shown that *B. pseudomallei* and *B. cenocepacia* have the ability to cause significant morphological changes in RAW 264.7 cell monolayers following infection (Utaisincharoen *et al*., 2001; 2004; Lamothe *et al*., 2007). To exclude the possibility that our inability to recover *B. mallei* from infected RAW 264.7 cells at an moi of 10 was due to monolayer destruction, fixed cells were examined using light and confocal microscopy at 24 h post infection. In comparison with control cells, monolayers infected with *B. mallei* at moi ranging from 0.1 to 10 appeared generally healthy and intact (Fig. 2A–C,E). Interestingly, sporadic multinucleated giant cell (MNGC) formation and evidence of actin-based motility were observed in monolayers infected at an moi of 1 but not at an moi of 10 (Fig. 2C–D). In stark contrast, monolayers infected with *B. pseudomallei* at an moi of 10 for control purposes, demonstrated significant MNGC formation and monolayer sloughing (Fig. 2F). Consistent with these observations, cytotoxicity assays also demonstrated that *B. pseudomallei* caused the RAW 264.7 cells to release significantly more lactate dehydrogenase (LDH) than *B. mallei* at all moi tested (Fig. 3). Taken together, these findings indicated that, in contrast to other closely related *Burkholderia* species, RAW 264.7 cells appeared to be capable of killing *B. mallei* when infected at a critical moi (≥ 10).

### *Burkholderia mallei* is a weak activator of RAW 264.7 cells

**Fig. 4:**
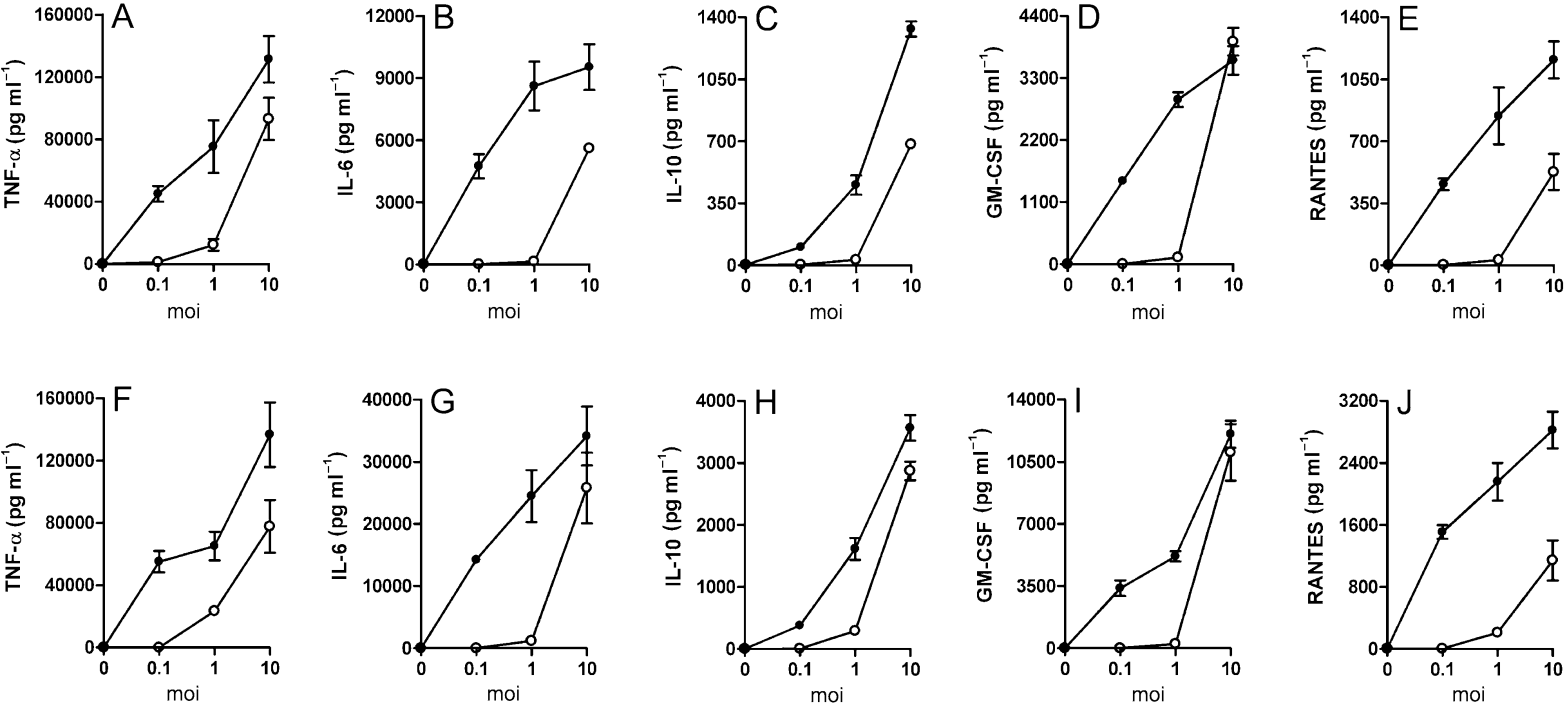
*B. mallei* is a weak activator of RAW 264.7 cells. Culture supernatants from monolayers infected with *B. mallei* (open circles) or *E. coli* (filled circles) at moi ranging from 0.1 to 10 were harvested at (A–E) 6 h or (F–J) 18 h post infection and assayed for the production of (A and F) TNF-α, (B and G) IL-6, (C and H) IL-10, (D and I) GM-CSF, and (E and J) RANTES. Values represent the means ± SD of three independent experiments assayed in duplicate.

Macrophages are known to play a critical role in the clearance of bacterial pathogens from host tissues. Upon activation, macrophages produce a variety of cytokines and chemokines that serve to regulate host defence mechanisms involved in these events (Collins and Kaufmann, 2001; Smith *et al*., 2001). To characterize the activation status of the *B. mallei*-infected RAW 264.7 monolayers, culture supernatants harvested at 6 and 18 h post infection were assayed for the production of a variety of pro- and anti-inflammatory cytokines and chemokines. At both 6 and 18 h, *B. mallei* was shown to be a relatively weak activator of the macrophages in comparison with *Escherichia coli* controls (Fig. 4A–J). In addition, although monolayers infected with *B. mallei* at moi of 1 and 10 produced significant amounts of TNF-α, only those infected at the higher moi of 10 were shown to produce high levels of IL-6, IL-10, GM-CSF and RANTES (Fig. 4A–J). Based upon these findings, a strong correlation appeared to exist between the activation status of the infected macrophages and their ability to clear intracellular *B. mallei*.

### INOS activity correlates with clearance of *B. mallei* from activated RAW 264.7 cells

**Fig. 5:**
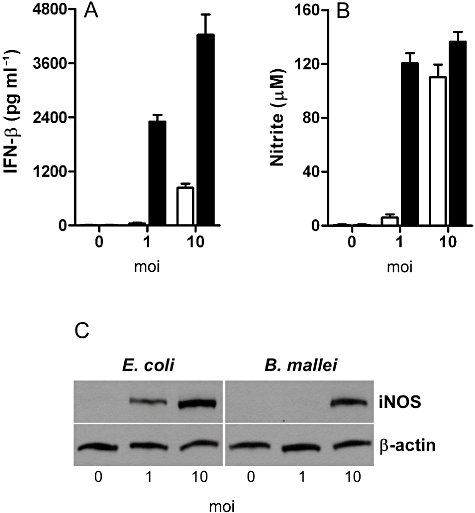
Production of IFN-β and NO by *B. mallei-* and *E. coli*-infected RAW 264.7 cells. Culture supernatants from monolayers infected with *B. mallei* (white bars) or *E. coli* (black bars) at moi of 1 and 10 were harvested (A) 6 h and (B) 24 h post infection and assayed for the production of IFN-β and NO respectively. Supernatants from mock-infected monolayers were included as negative controls. Values represent the means ± SD of three independent experiments assayed in duplicate or triplicate. C. Cell lysates prepared from *E. coli*-infected and *B. mallei*-infected monolayers harvested 24 h post infection were assayed for the presence of iNOS by immunoblot analysis. β-actin was included as a loading control. Results are representative of three independent experiments.

IFN-β is an important modulator of innate host defence responses. For example, by acting in an autocrine fashion, IFN-β can augment bactericidal effector mechanisms by enhancing iNOS expression in activated macrophages (Thomas *et al*., 2006). Interestingly, studies have shown that, following infection with *B. pseudomallei*, IFN-β is only weakly produced by RAW 264.7 cells (Utaisincharoen *et al*., 2004). To examine the ability of *B. mallei* to stimulate RAW 264.7 cells to produce this cytokine, culture supernatants harvested at 6 h post infection were assayed for IFN-β by ELISA. Consistent with the results described in Fig. 4, *B. mallei* was shown to be a relatively weak activator of IFN-β production in comparison with *E. coli*. Furthermore, monolayers were only shown to produce significant levels of this cytokine when infected at an moi of 10 (Fig. 5A).

Production of ROS and RNOS is known to be important for the killing of a variety of pathogens by activated macrophages. Previous studies have demonstrated that RNOS are important in the control of infections caused by *B. pseudomallei* and *B. cepacia* (Miyagi *et al*., 1997; Saini *et al*., 1999). Studies have shown, however, that *B. pseudomallei* appears to be able to interfere with iNOS expression in RAW 264.7 cells (Utaisincharoen *et al*., 2001; 2004). To assess the ability of *B. mallei* to stimulate RAW 264.7 cells to produce NO, culture supernatants harvested at 24 h post infection were assayed for nitrite using Griess reagent. Unlike the results observed for the *E. coli* controls, high levels of NO were only produced by monolayers infected with *B. mallei* at an moi of 10 (Fig. 5B). Consistent with these observations, immunoblot analyses of cell lysates prepared from *E. coli-* and *B. mallei*-infected monolayers demonstrated a strong correlation between iNOS expression and NO production (Fig. 5C).

**Fig. 6:**
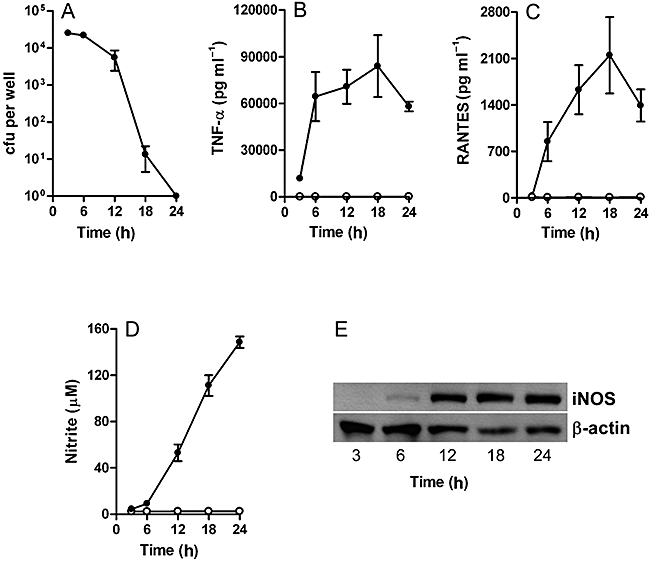
Clearance of *B. mallei* from activated RAW 264.7 macrophages correlates with iNOS activity. Monolayers were infected with *B. mallei* at an moi of 10. A. Bacterial loads were determined at 3, 6, 12, 18 and 24 h post infection. Values represent the means ± SD of three independent experiments. Culture supernatants harvested at 3, 6, 12, 18 and 24 h from mock-infected (open circles) and *B. mallei*-infected (filled circles) monolayers were assayed for the production of (B) TNF-α, (C) RANTES, and (D) NO. Values represent the means ± SD of three independent experiments assayed in duplicate or triplicate. E. Cell lysates prepared from *B. mallei*-infected monolayers harvested at 3, 6, 12, 18 and 24 h post infection were assayed for the presence of iNOS by immunoblot analysis. β-actin was included as a loading control. Results are representative of three independent experiments.

To further examine the importance of iNOS activity with regards to *B. mallei* killing, experiments were conducted in an attempt to correlate bacterial survival with RAW 264.7 cell activation, iNOS expression and NO production. Following infection of the monolayers at a critical moi of 10, bacterial survival was shown to steadily decrease to zero over a 24 h period (Fig. 6A). By monitoring TNF-α and RANTES levels, macrophages were shown to be highly activated over the course of the assay (Fig. 6B and C). In addition, NO levels were also shown to increase in a linear fashion over the 24 h period (Fig. 6D). Consistent with these results, immunoblot analysis of iNOS levels correlated with both NO production and bacterial killing (Fig. 6E). Taken together, these findings indicated that when infected at a critical moi, iNOS-dependent mechanisms appeared to be important in the clearance of intracellular *B. mallei* from RAW 264.7 cells.

### Inhibition of iNOS activity facilitates survival of *B. mallei* in activated RAW 264.7 cells

**Fig. 7:**
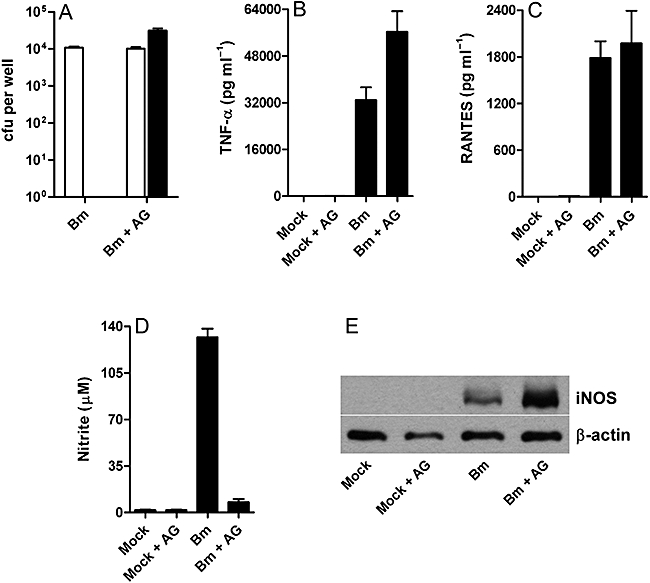
Inhibition of iNOS activity facilitates survival of *B. mallei* in activated RAW 264.7 cells. Monolayers infected with *B. mallei* (Bm) at an moi of 10 were incubated in the absence or presence of AG (200 μg ml^−1^) at 1 h post infection. A. Uptake (white bars) and intracellular survival (black bars) were determined at 3 and 24 h post infection respectively. Values represent the means ± SD of three independent experiments. Culture supernatants harvested at 24 h post infection from mock-infected and *B. mallei*-infected monolayers were assayed for the production of (B) TNF-α, (C) RANTES and (D) NO. Values represent the means ± SD of three independent experiments assayed in duplicate or triplicate. E. Cell lysates prepared from mock-infected and *B. mallei*-infected monolayers harvested at 24 h post infection were assayed for the presence of iNOS by immunoblot analysis. β-actin was included as a loading control. Results are representative of three independent experiments.

To confirm that iNOS activity was important for the clearance of intracellular *B. mallei* from RAW 264.7 cells, modified kanamycin-protection assays, using the iNOS inhibitor aminoguanidine (AG), were performed at an moi of 10. Results of these assays demonstrated that, in comparison with controls, treatment with AG (200 μg ml^−1^) appeared to have little impact on bacterial uptake (Fig. 7A). Quantification of intracellular bacteria at 24 h post infection also demonstrated that *B. mallei* was cleared by activated macrophages in the absence of AG. However, in the presence of AG, survival was shown to increase >10 000-fold over control levels (Fig. 7A). To confirm that AG did not inhibit macrophage activation, culture supernatants were assayed for pro-inflammatory cytokine and chemokine production. Results of these analyses demonstrated that, whether incubated in the presence or absence of AG, infected monolayers produced significantly higher levels of TNF-α and RANTES than the mock-infected cells (Fig. 7B and C). Consistent with predicted results, high levels of NO were only produced by infected monolayers in the absence of AG (Fig. 7D). Importantly, immunoblot analyses of cell lysates confirmed that the decreases in NO production were not due to reduced iNOS expression, but due to the inhibition of iNOS activity by the AG (Fig. 7E). Based upon these findings, iNOS activity appeared to be critical for the clearance of *B. mallei* by activated RAW 264.7 cells.

### Lipopolysaccharide (LPS) stimulates the clearance of *B. mallei* from RAW 264.7 cells

**Fig. 8:**
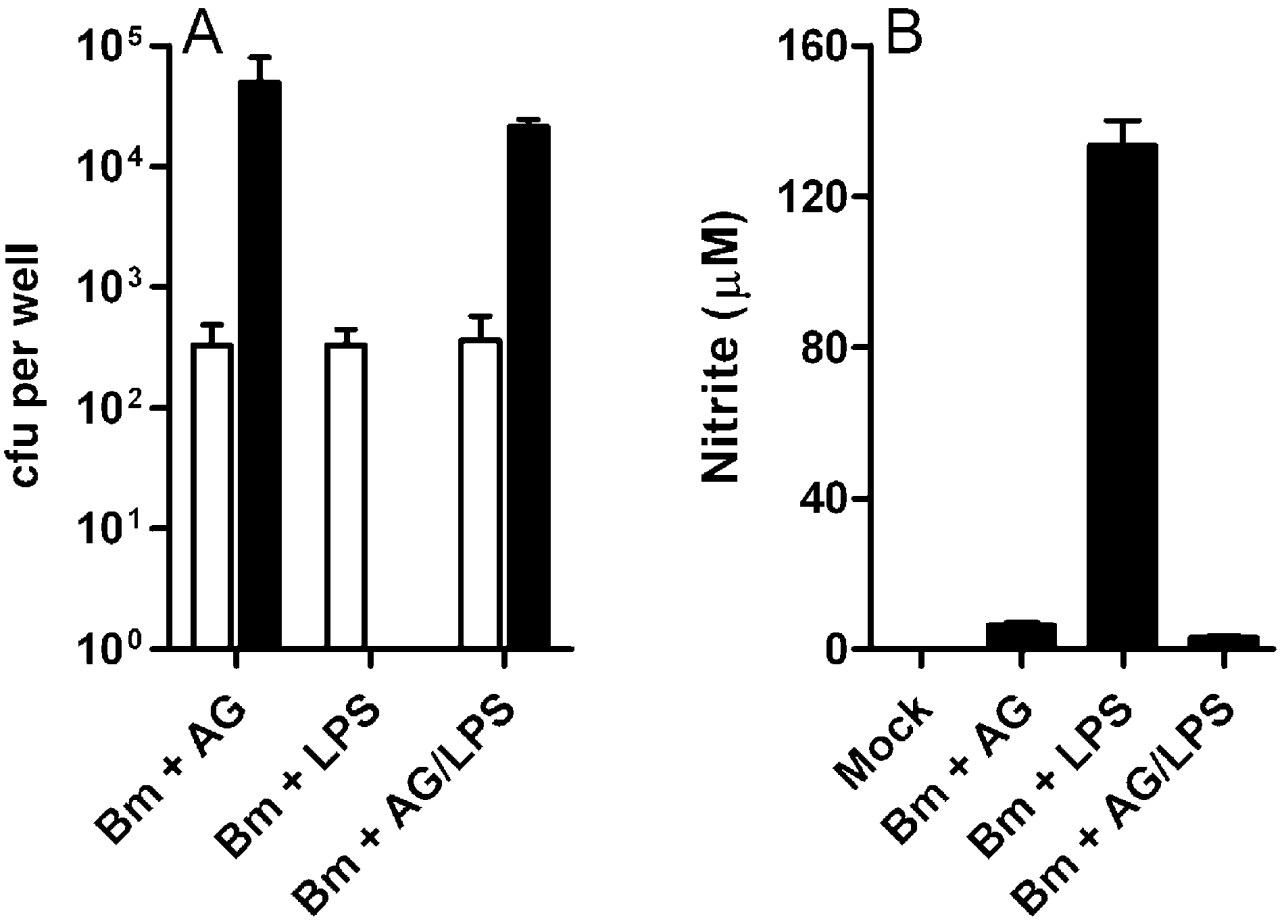
*E. coli* LPS stimulates the clearance of *B. mallei* from RAW 264.7 cells infected at an moi of 1. Infected monolayers were incubated with either AG (200 μg ml^−1^), LPS (100 ng ml^−1^), or AG (200 μg ml^−1^) and LPS (100 ng ml^−1^) at 1 h post infection. A. Uptake (white bars) and intracellular survival (black bars) were determined at 3 and 24 h post infection respectively. Values represent the means ± SD of three independent experiments. B. Culture supernatants harvested 24 h post infection from mock-infected and *B. mallei*-infected monolayers were assayed for the production of NO. Values represent the means ± SD of three independent experiments assayed in triplicate.

Toll-like receptor (TLR) signalling is an important means by which phagocytic cells are activated by PAMPs (Carpenter and O'Neill, 2007). Numerous studies have demonstrated that *E. coli* LPS is a potent activator of murine macrophages (Weisz *et al*., 1994; Zughaier *et al*., 2005; Okemoto *et al*., 2006). In particular, RAW 264.7 cells stimulated with *E. coli* LPS are known to produce high levels of NO (Weisz *et al*., 1994; Zughaier *et al*., 2005). To confirm the relationship between activated RAW 264.7 cells, iNOS activity and bacterial clearance, monolayers infected with *B. mallei* at an moi of 1 were incubated with various combinations of AG (200 μg ml^−1^) and LPS (100 ng ml^−1^). Results of these assays demonstrated that uptake levels did not appear to be influenced by the AG and LPS treatments (Fig. 8A). Quantification of intracellular survival at 24 h post infection demonstrated that *B. mallei* was cleared by RAW 264.7 cells treated with LPS alone. In contrast, *B. mallei* was shown to survive in AG-treated macrophages even in the presence of LPS (Fig. 8A). In order to correlate intracellular survival with iNOS activity, culture supernatants harvested at 24 h post infection were assayed for NO production. Consistent with predicted results, high levels of NO were only produced by infected monolayers in the absence of AG (Fig. 8B). Taken together, these findings indicated that when infected at a subcritical moi (≤ 1), RAW 264.7 cells could be stimulated by LPS to clear *B. mallei* in an iNOS-dependent manner.

## Discussion

The ability of *B. mallei* to persist within professional phagocytic cells is thought to be an important means by which this organism evades specific host immune responses. Previous reports have shown that *B. mallei* is able to survive and replicate within J774.2 macrophages and that these phenotypes are dependent upon the expression of the Bsa T3SS (Ribot and Ulrich, 2006). In the present study, we characterized the interactions of *B. mallei* with RAW 264.7 murine macrophages and demonstrated that moi significantly affected the outcome of these events. Utilizing modified kanamycin-protection assays, initial studies demonstrated that *B. mallei* was internalized in an moi-dependent fashion. However, it appeared that the uptake levels for this organism were significantly lower (10- to 100-fold) than those typically observed in our laboratory for *B. pseudomallei* as well as those previously described for *B. cenocepacia* (P.J. Brett and F.C. Gherardini, unpublished; Lamothe *et al*., 2007). At present, the reason for this phenomenon remains unclear. Recent findings in our laboratory, however, suggest that flagella may play an important role in the uptake of pathogenic *Burkholderia* species by RAW 264.7 cells. In particular, *B. pseudomallei* motility mutants have been shown to exhibit reduced uptake in comparison to parental strains (P.J. Brett and F.C. Gherardini, unpublished). Consistent with these observations, studies by Inglis *et al*. (2003) have also demonstrated that the flagellar apparatus expressed by *B. pseudomallei* assists entry into *Acanthamoeba astronyxis* trophozoites.

Previous studies have demonstrated that several pathogenic *Burkholderia* species are able to persist in a variety of human and murine cell types, and that significant cellular abnormalities are often observed upon infection with these organisms (Jones *et al*., 1997; Miyagi *et al*., 1997; Harley *et al*., 1998; Saini *et al*., 1999; Kespichayawattana *et al*., 2000; Utaisincharoen *et al*., 2001; Lamothe *et al*., 2007). *B. pseudomallei* and *B. mallei*, for instance, have been shown to cause MNGC formation both *in vitro* and *in vivo* (Duval and White, 1907; Harley *et al*., 1998; Kespichayawattana *et al*., 2000). This unique phenomenon is thought to be due in part to the actin-based intra- and intercellular motility phenotypes displayed by these pathogens (Harley *et al*., 1998; Kespichayawattana *et al*., 2000; Stevens *et al*., 2002; Breitbach *et al*., 2003). *B. cenocepacia*, in addition, is known to persist within phagocytic vacuoles and cause a variety of morphological changes in RAW 264.7 cells, including rounding, filamentation and the development of highly dense cytoplasms (Saini *et al*., 1999; Lamothe *et al*., 2007). In the present study, *B. mallei* was shown to survive and replicate in RAW 264.7 cells only when infected at moi of 0.1 and 1, but not at an moi of 10. As demonstrated by light microscopy and cytotoxicity assays, these results could not simply be explained by macrophage destruction because the monolayers infected at the moi of 10 were seemingly healthy and intact. Based upon these findings, it appears that, in comparison with other pathogenic *Burkholderia* species, *B. mallei* demonstrates rather unique survival characteristics in RAW 264.7 cells. Studies are ongoing to determine whether or not this clearance phenotype is specific to RAW 264.7 cells.

In this study, we demonstrated that *B. mallei* is a relatively weak activator of RAW 264.7 cells in comparison with *E. coli* controls. Results indicated that on an moi basis, 10- to 100-fold more *B. mallei* than *E. coli* were required to stimulate infected monolayers to produce similar levels of cytokines and chemokines. At present the reasons for these findings are unclear. Studies in our laboratory, however, indicate that this may be an LPS-related phenomenon. Recently, we demonstrated that *B. mallei* expresses complex mixture of tetra- and penta-acylated lipid A species that are similar in structure to those expressed by other pathogenic *Burkholderia* species (De Soyza *et al*., 2004; Silipo *et al*., 2005; Brett *et al*., 2007). In addition, we demonstrated that *B. mallei* LPS was a potent activator of human TLR4 complexes and stimulated THP-1 cells, U937 cells as well as human monocyte-derived macrophages and dendritic cells to produce high levels of TNF-α, IL-6 and RANTES (Brett *et al*., 2007). Interestingly, preliminary studies in our laboratory suggest that *B. mallei* LPS, like *B. pseudomallei* LPS, is a less potent activator of RAW 264.7 cells than *E. coli* LPS (P.J. Brett and F.C. Gherardini, unpublished; Utaisincharoen *et al*., 2000). To what extent LPS influences the ability of *B. mallei* to stimulate RAW 264.7 cells remains to be experimentally defined. In addition, studies are ongoing to determine why RAW 264.7 cells appear to be less responsive to *B. mallei* LPS than human tissue cell lines and primary phagocytic cells.

Interferons are pro-inflammatory cytokines involved in both innate and acquired immune responses. One of the most important functions of these cytokines appears to be the sensitization of macrophages to PAMPs (Takaoka and Yanai, 2006). Additionally, both type I (IFN-β) and type II (IFN-γ) interferons are known to enhance iNOS expression by macrophages (Boehm *et al*., 1997; MacMicking *et al*., 1997; Rottenberg *et al*., 2000). Previous studies have shown that many pathogenic *Burkholderia* species are rapidly killed by IFN-γ-activated macrophages (Miyagi *et al*., 1997; Utaisincharoen *et al*., 2001). The molecular mechanism(s) by which these organisms are cleared, however, appear to greatly depend upon the lineage of the macrophage. Reports by Miyagi *et al*. (1997) have demonstrated that J774.1 murine macrophage-like cells can be activated with IFN-γ to clear intracellular *B. pseudomallei* and that optimal killing by these cells appeared to rely upon the presence of both ROS and RNOS. Similarly, studies by Utaisincharoen *et al*. (2001; 2003; 2004) have shown that *B. pseudomallei* can be cleared by IFN-γ-activated RAW 264.7 cells and that iNOS activity is important for bacterial killing. However, recent studies by Breitbach *et al*. (2006) have demonstrated that IFN-γ-activated C57BL/6 bone marrow-derived macrophages are capable of killing *B. pseudomallei* in an iNOS-independent manner. Based upon these findings, it appears likely that pathogenic *Burkholderia* species may be cleared from activated macrophages via both RNOS-dependent and RNOS-independent mechanisms.

IFN-β is a dominant factor in the shaping of innate and adaptive immune responses. Soon after activation, IFN-β produced by macrophages mediates target gene expression via the activation of JAK-STAT signalling pathways (Theofilopoulos *et al*., 2005). Importantly, IFN-β has been shown to be a necessary component of signalling processes leading to the induction of iNOS gene expression in murine macrophages (Jacobs and Ignarro, 2001; Thomas *et al*., 2006). In the present study, we demonstrated that clearance of *B. mallei* by activated RAW 264.7 cells strongly correlated with both IFN-β production and iNOS activity. In addition, time-course assays demonstrated that the kinetics of *B. mallei* killing by activated RAW 264.7 cells correlated directly with the kinetics of NO production. These results, in combination with those obtained from the AG inhibition assays, suggested a critical role for iNOS activity and RNOS production in clearance of *B. mallei* from activated RAW 264.7 cells. At present, whether or not RNOS produced by activated RAW 264.7 cells function synergistically with other macrophage effector species (i.e. ROS) to clear *B. mallei* remains to be defined. It also remains to be determined why infection of RAW 264.7 cells with *B. mallei* resulted in high levels of iNOS expression/activity compared with other pathogenic *Burkholderia* species (Saini *et al*., 1999; Utaisincharoen *et al*., 2001; 2004).

Toll-like receptors are a family of PRRs that are important in the development of both innate and adaptive immune responses. All TLRs activate a common signalling pathway that results in the activation of nuclear factor-kappa B transcription factors, mitogen-activated protein kinases, p38 and JNK (Barton and Medzhitov, 2003). TLRs are activated in response to a variety of PAMPs expressed by bacteria, viruses, fungi and protozoa. Following recognition of PAMPs, a cascade of intracellular signalling events is activated, which results in the stimulation of pro-inflammatory cytokines such as TNF-α and IL-6 (Miggin and O'Neill, 2006). Additionally, production of type I interferons may be induced via the stimulation of a number of TLRs, including TLR4 (Miggin and O'Neill, 2006). Mounting evidence suggests that TLR agonists have significant potential in the treatment of a number of disease conditions (Pandey and Agrawal, 2006). Recently, Waag *et al*. (2006) demonstrated that BALB/c mice could be protected from an aerosol challenge of *B. mallei* using the TLR9 agonist CpG ODN 7909. In the present study, we have demonstrated that macrophages infected with *B. mallei* at a subcritical moi could be stimulated with the prototypic TLR4 agonist, LPS, to clear intracellular bacteria. In addition, it was shown that clearance of *B. mallei* from infected RAW 264.7 cells correlated strongly with iNOS activity, supporting the prediction that RNOS are important for killing this organism. Studies are ongoing to more fully characterize the role of TLR signalling in the clearance of *B. mallei* from RAW 264.7 cells.

In summary, we have demonstrated that iNOS activity is critical for the clearance of *B. mallei* from activated RAW 264.7 cells. In addition, we have shown that macrophages infected with *B. mallei* at a subcritical moi can be stimulated by LPS to kill the organism in an iNOS-dependent manner. In order to more fully characterize the importance of specific macrophage effector mechanisms against this organism, however, experiments utilizing primary macrophages derived from a variety of wild-type and transgenic mice will need to be conducted. Studies are ongoing to address this as well as examine the interactions of *B. mallei* with human and equine macrophages. Future studies are also planned to investigate reasons underlying the differential survival phenotypes of *B. pseudomallei* and *B. mallei* within RAW 264.7 cells.

## Experimental procedures

### Bacterial strains, growth conditions and reagents

*Escherichia coli* HB101 was obtained from the American Type Culture Collection (ATCC). *B. mallei* ATCC 23344 (Yabuuchi *et al*., 1992) and *B. pseudomallei* DD503 (Moore *et al*., 1999) were kindly provided by Dr Donald Woods (University of Calgary). *E. coli* was grown at 37°C on LB-Lennox (LBL; Difco) agar or in LBL broth. *B. mallei* and *B. pseudomallei* were grown at 37°C on LBL agar or in LBL broth supplemented with 4% glycerol. Brucella agar (Difco) supplemented with 4% glycerol (BB4G) was used for plate counts and sterility analyses. For macrophage assays, bacteria were subcultured 1:50 into LB4G broth from overnight cultures and grown at 37°C for 3–4 h. All studies utilizing viable *B. mallei* and *B. pseudomallei* were conducted under biosafety level 3 containment. Unless stated otherwise, all reagents were purchased from Sigma. *E. coli* O55:B5 LPS was extracted four times with 90% EtOH to remove any contaminating phospholipids prior to use. LPS stocks were prepared in pyrogen-free water (Cambrex) and quantified on a w/v basis.

### Cell culture

The murine macrophage cell line RAW 264.7 (ATCC TIB-71) was obtained from ATCC. The cells were maintained in Dulbecco's modified Eagle's medium (DMEM; Invitrogen) supplemented with 10% (v/v) heat-inactivated fetal bovine serum (FBS; Invitrogen) and a standard mixture of antibiotics (100 U ml^−1^ penicillin, 100 μg ml^−1^ streptomycin and 250 μg ml^−1^ amphotericin B) at 37°C under an atmosphere of 5% CO_2_. For macrophage survival assays, RAW 264.7 cells resuspended in DMEM supplemented with FBS (DMEM-10) were transferred into 24-well tissue culture plates at a density of ∼1 × 10^6^ cells per well and incubated overnight.

### Macrophage survival assays

Bacterial uptake and survival were quantified utilizing modified kanamycin-protection assays. Bacteria grown to early log phase were pelleted and resuspended at various cell densities in DMEM-10. The bacterial suspensions were then added in triplicate onto RAW 264.7 cells at moi ranging from 0.1 to 10. The monolayers were incubated with the bacteria at 37°C under an atmosphere of 5% CO_2_ for 1 h and then washed twice with Hanks' Balanced Salt Solution (HBSS; Invitrogen) to remove extracellular bacteria. Infected RAW 264.7 cells were then incubated in fresh DMEM-10 containing 250 μg ml^−1^ kanamycin (DMEM-10K) to suppress the growth of residual extracellular bacteria. At various time points post infection, monolayers were lysed with 0.25% (v/v) Triton X-100, and serial dilutions of the lysates were plated onto BB4G agar and incubated at 37°C for 48 h. Plate counts were then used to enumerate bacterial loads. Uptake and intracellular survival were routinely quantified at 3 and 24 h post infection. For time-course experiments, uptake and intracellular survival were quantified at 3, 6, 12, 18 and 24 h respectively.

### Light microscopy

Light micrographs of RAW 264.7 monolayers infected with *B. mallei* or *B. pseudomallei* at an moi of 10 were obtained at 24 h post infection. Monolayers were washed twice with phosphate-buffered saline (PBS) and fixed with 2.5% paraformaldehyde for 15 min at room temperature prior to use. Fixed monolayers were visualized with a Nikon Eclipse TE300 (Nikon Instruments) inverted microscope using a 10× objective.

### Immunofluorescence staining and confocal microscopy

RAW 264.7 cells were grown overnight on 12 mm glass coverslips (Fisher Scientific) and infected at an moi of 1 using similar approaches to those described for the macrophage survival assays. Monolayers were immunostained at room temperature essentially as previously described (Knodler *et al*., 2003). Briefly, at 24 h post infection, monolayers were fixed in 2.5% paraformaldehyde for 15 min, followed by extensive washing in PBS. Cells were then permeabilized in PBS containing 10% normal goat serum (Invitrogen) and 0.1% (w/v) saponin (SS-PBS) for 20 min. Cells were incubated with rabbit polyclonal antiserum raised against formalin-fixed *B. thailandensis* diluted 1:50 in SS-PBS for 45 min, washed several times in PBS/0.05% (w/v) saponin, and then incubated with Alexa Fluor® 568 goat α-rabbit IgG (1:800; Invitrogen), Alexa Fluor® 488 phalloidin (1:200, Invitrogen) and DRAQ5™ (1:1000, Alexis Biochemicals) in SS-PBS for a further 45 min. Following extensive washing in PBS, coverslips were mounted onto glass slides using Mowiol. Laser confocal microscopy was performed with a Zeiss 510 META confocal imaging system equipped with a Ar, HeNe laser on an inverted Axiovert 200 M microscope using a 63× oil objective (Carl Zeiss MicroImaging). Images of 1024 × 1024 pixels were acquired using Zeiss 510 META software (Carl Zeiss MicroImaging).

### Cytotoxicity assays

Culture supernatants were harvested from infected monolayers at 24 h and sterilized using 0.45 μm Millex™ syringe driven filter units (Millipore Corporation). Aliquots of each were plated onto BB4G agar and incubated at 37°C for at least 72 h to confirm sterility. The sterilized supernatants were then assayed for LDH release using a CytoTox 96® Non-Radioactive Cytoxicity Assay kit (Promega). Maximum release was achieved by lysis of monolayers with Triton X-100 at a final concentration of 1% (v/v). LDH released by uninfected cells was designated as spontaneous release. Cytotoxicity was calculated as follows: % cytotoxicity = (test LDH release − spontaneous release)/(maximal release − spontaneous release).

### Cytokine, chemokine and NO assays

Culture supernatants were harvested from infected monolayers at various time points and sterilized using 0.45 μm Millex™ syringe driven filter units (Millipore Corporation). Aliquots of each were plated onto BB4G agar and incubated at 37°C for at least 72 h to confirm sterility. The sterilized supernatants were then assayed for the production of TNF-α, IL-6, IL-10, GM-CSF and RANTES using murine SearchLight Custom Multiplex Arrays (Pierce). IFN-β levels were also determined using a Mouse IFN-Beta ELISA kit (R & D Systems). NO production was estimated from nitrite levels using a Nitric Oxide Assay Kit (Pierce).

### INOS immunoblot analyses

Infected monolayers were washed three times with HBSS, and cells were lysed with 150 μl of ice-cold 1× RIPA Buffer (Pierce) supplemented with 1× HALT™ Protease Inhibitor Cocktail (Pierce). The lysates were then mixed with an equal volume of 2× Tris-glycine SDS sample buffer (Invitrogen) and heated to 100°C for 10 min. Aliquots of each were plated onto BB4G agar and incubated at 37°C for at least 72 h to confirm sterility. Sterilized lysates were electrophoresed on 10% Tris-glycine gels (Invitrogen), and proteins were electrophoretically transferred to nitrocellulose membranes (0.45 μm pore size; Invitrogen). Immunoblot analyses were performed at room temperature. Membranes were blocked with Starting Block™ Blocking Buffer (Pierce) for 15 min and then incubated for 1 h with a 1:2000 dilution of an anti-iNOS mAb (clone NOS-IN) in TBS-T [20 mM Tris-HCl (pH 7.5), 500 mM NaCl and 0.05% Tween 20]. Membranes were then washed three times with TBS-T and incubated for 1 h with a 1:5000 dilution of an anti-mouse IgG peroxidase conjugate in TBS-T. Membranes were washed three more times with TBS-T, following which the blots were visualized using ECL™ Plus Western Blotting Detection Reagents (GE Healthcare). To confirm equal loading of the cell lysates, membranes were stripped with Restore™ Western Blot Stripping Buffer (Pierce) and re-probed using a 1:4000 dilution of an anti-β-actin mAb (clone AC-15) and a 1:10000 dilution of the anti-mouse IgG peroxidase conjugate.

### INOS inhibition and LPS stimulation assays

iNOS inhibition and LPS stimulation assays were performed using similar approaches to those described for the macrophage survival assays. For iNOS inhibition assays, RAW 264.7 cells were infected with *B. mallei* at an moi of 10. The monolayers were incubated with the bacteria for 1 h and then washed twice with HBSS. Infected monolayers were then incubated for a further 2 or 23 h in either DMEM-10K or DMEM-10K containing 200 μg ml^−1^ AG. For the LPS stimulation assays, RAW 264.7 cells were infected with *B. mallei* at an moi of 1. The monolayers were incubated with the bacteria for 1 h and then washed twice with HBSS. Infected monolayers were then incubated for a further 2 or 23 h in DMEM-10K containing 200 μg ml^−1^ AG, DMEM-10K containing 100 ng ml^−1^ LPS, or DMEM-10K containing 200 μg ml^−1^ AG and 100 ng ml^−1^ LPS.
